# Pelvic floor disorder and relevant factors in Iranian women of reproductive age: a cross-sectional study

**DOI:** 10.1186/s12905-023-02226-1

**Published:** 2023-02-16

**Authors:** Fatemeh Rashidi, Mojgan Mirghafourvand

**Affiliations:** 1 Students’ Research Committee, Nursing and Midwifery Faculty, Tabriz University of Medical Sciences, Tabriz, Iran; 2 Social Determinants of Health Research Center, Faculty of Nursing and Midwifery, Tabriz University of Medical Sciences, Shariati Street, P.O. Box: 51745-347, Tabriz, 513897977 Iran

**Keywords:** Pelvic floor disorder, Urinary incontinence, Anal incontinence, Pelvic organ prolapse

## Abstract

**Background:**

With high severity and prevalence, pelvic floor disorder is a health issue that women face worldwide. Different demographic-obstetric factors are involved in the emergence of this dysfunction that can have many adverse effects on a woman’s quality of life. Hence, this study aimed to determine the prevalence of pelvic floor disorder and its related socio-demographic and obstetric factors among Iranian women of reproductive age.

**Methods:**

The statistical population of this cross-sectional study included 400 woman of reproductive age (15–49 years) covered by the health centers of Tabriz, Iran in 2022. The cluster sampling method was employed to select the participants. The data collection tools were a socio-demographic and obstetric characteristics questionnaire and the PDFI-20 (Pelvic Floor Distress Inventory-20). The chi-squared test was conducted to determine the association between socio-demographic and obstetric characteristics and prevalence of pelvic floor disorder in a bivariate analysis, whereas the multivariate logistic regression test was used in a multivariate analysis.

**Results:**

The general prevalence of pelvic floor disorder was 76%. The prevalence rates of pelvic organ prolapse distress 6 (POPDI-6), colorectal-anal distress 8 (CRAD-8), and urinary distress (UDI-6) were 54.3%, 61.8%, and 49.3%, respectively. The results of the multivariate logistic regression test indicated that constipation (odds ratio = 5.62; 95% CI 1.97 to 16.03; *P* = 0.001) increased the risk of pelvic floor disorder.

**Conclusions:**

According to the findings, the prevalence of pelvic floor disorder is high among Iranian women of reproductive age. This condition is correlated with constipation. Therefore, screening is recommended through valid tools in addition to offering preventive measures such as preventing and curing constipation to reduce the risk of pelvic floor disorder.

## Background

Pelvic floor disorder is a seriously prevalent health issue among women [1] caused by damage to pelvic floor muscles and pelvic fascia [2]; this condition includes a wide variety of dysfunctions such as colorectal distress, bladder dysfunction, pelvic organ prolapse, and sexual dysfunction [3]. These dysfunctions manifest different symptoms such as the inability to control bladder (i.e*.*, urinary incontinence) [4], the inability to control fecal excretion (i.e., loose and hard stools) [5], and bulge or heaviness caused by a sag in vaginal walls [6]. Pelvic floor disorder can considerably have adverse effects on the quality of life and a women’s perception of her image [7, 8]. Not only does this disorder affect physical aspects of a woman’s life, but it also impacts social and psychological aspects seriously. In fact, women with pelvic floor disorder are prone to anxiety and depression [9].

According to different studies, the worldwide prevalence of pelvic floor disorder ranges between 12 and 40% [10, 11]. It was reported 42% in Iran by a study in 2012 [12]. Pregnancy and childbirth are the most important factors in weakening pelvic floor muscles due to physiological and hormonal changes [13, 14]. In fact, pelvic floor disorder is considered a multifactor disorder [15]. Epidemiological studies indicate that age, constipation, obesity, menopause, parity, and childbirth type can affect the emergence of this dysfunction [1, 14]. Recently, a review study has introduced genes as an effective factor in the emergence of pelvic floor disorder, especially pelvic organ prolapse distress [16]. The highest rate of pelvic floor disorders reported after vaginal delivery is related to urinary incontinence (28%) and pelvic organ prolapse (14%) [17]. Nevertheless, there is no evidence regarding the role of caesarean section in protecting pelvic floor muscles to prevent the potential occurrence of pelvic floor disorder [18].

Although women are advised to do postnatal exercises to prevent and cure pelvic floor disorder, the protective effects of these exercises have not been proved yet [19]. Moreover, in a review have introduced physical therapy as an effective method for improving the symptoms of pelvic floor disorder caused by pregnancy. Overall, more than 20% of women need surgical operations to treat bladder dysfunction and pelvic organ prolapse distress [20].

Reportedly, the elderly population will double worldwide by 2050. Since aging and menopause are two risk factors in pelvic floor disorder, the prevalence of this condition is estimated to increase more than ever before [20]. Given the complications of pelvic floor disorder and its disruptive effects on women’s quality of life [8], determining its prevalence and risk factors can have a key role in offering strategies for prevention and timely treatment of this condition. Hence, this study aimed to determine the prevalence of pelvic floor disorder and its relevant socio-demographic and obstetric factors.

### Research questions

- What is the prevalence of pelvic floor disorders among Iranian women of reproductive age?
- What socio-demographic and obstetric factors are related to pelvic floor disorder among Iranian women of reproductive age?

## Methods

### Study design and participants

The statistical population of this descriptive-analytic cross-sectional study included 400 non-pregnant or reproductive age (15–49 years) visiting health centers of Tabriz, Iran from October 2021 to December 2022.

The inclusion criteria were as follows: being of the reproductive age (15–49 years), being married and having no pregnancy diagnosed during research. Furthermore, the exclusion criteria were as follows: having a history of recent urinary tract infection, having a history of gynecological surgery (e.g., reconstructive and cosmetic surgeries), taking tricyclic antidepressants (e.g., amitriptyline, clomipramine, dosulepin, doxepin, imipramine, lofepramine, nortriptyline, and trazodone), selective serotonin reuptake inhibitors (e.g*.*, citalopram, fluoxetine, paroxetine, and sertraline), monoamine oxidase (e.g., phenelzine and tranylcypromine), and reuptake inhibitor of serotonin and noradrenaline (e.g., reboxetine and venlafaxine), experiencing any stressful events (e.g., divorce, death of a first-degree relative, and diagnosis of an incurable disease for a first-degree relative in the past three months), and experiencing a childbirth in the past six months.

### Sample size

The sample size was determined nearly 191 participants with an approximate prevalence of 42% [12] through *n* = z^2^pq/d^2^ with a reliability of 95% and an accuracy of 0.07. The final sample size was calculated 400 with considering the design effect of 2 due to using of cluster sampling method and the probable attrition of 5%.

### Sampling

After a permit was granted by the Ethics Committee of Tabriz University of Medical Sciences (IR.TBZMED.REC.1400.1073), sampling was allowed through introduction letters to health centers of Tabriz Province, Iran. The research environment included the health centers of Tabriz, in which cluster sampling was administered. First, 23 health centers were randomly selected from 92 centers through www.random.org website. The researcher then visited the designated centers to extract the lists of women of reproductive age meeting the inclusion criteria from SIB system (Integrated health system in Iran). The women were then selected from each center through proportional to size sampling method. They were analyzed via phone calls in terms of inclusion and exclusion criteria. They were also asked whether they had taken U/A (urine analysis) tests in the past three days. The participants who had not taken U/A tests in the past three days were asked about the urinary tract infection symptoms. If they did not have any symptoms and met the inclusion criteria, they were provided with the necessary information regarding the study, implementation steps, and confidentiality of data. They were then offered to participate in the study. If they agreed on participation, they were asked to be present at a health center at a specific time. Upon an in-person visit, the participants were asked to sign the informed consent forms and to complete the data collection tools. Out of 400 eligible participants, only one received the link to electronic questionnaires via WhatsApp (due to absence for her hectic schedule).

### Data collection tools

The research tools were a socio-demographic and obstetric characteristics questionnaire and the Pelvic Floor Distress Inventory-20 (PDFI-20).

### Socio-demographic and obstetric characteristics questionnaire

This questionnaire was employed to collect information on age, body mass index (BMI), education, occupation, economic status, sports exercises, cigarette smoking, medical history (e.g*.*, chronic respiratory conditions, diabetes, hypertension, and constipation), and obstetric history (e.g*.*, the number of pregnancies and deliveries, childbirth type, and history of gynecological surgeries).

### Pelvic floor distress inventory-20 (PDFI-20)

This 20-item tool was designed by Barber et al*.* (2005) [21] to evaluate and classify pelvic floor disorder as three categories called pelvic organ prolapse distress (6 items), colorectal-anal distress (8 items), and urinary distress (6 items). Each item is scored from 1 to 4. If a response is “no”, the score is 0. If a response is “yes”, the score will be given from 1 to 4 (0 = “nothing”, 1 = “not at all”, 2 = “to some extent”, 3 = “average”, and 4 = “very high”). The scores of each scale will be determined by calculating the average score of each category multiplied by 25. The maximum score of each scale (0–100) will be calculated by adding the scores of three scales to the total score (0–300). The higher the score, the more effective the pelvic floor disorder. The validity and reliability of this questionnaire were confirmed by Rashidi et al*.* (2022) in an Iranian population. The reliability of this questionnaire was confirmed 0.84 through Cronbach’s alpha and Intraclass Correlation Coefficient (ICC) equal to 0.98. Its article is in the peer reviewing stage.

### Data analysis

Data analysis was performed in SPSS 26 through descriptive statistics such as frequency (percentage), mean, and standard deviation (SD) to describe the socio-demographic and obstetric characteristics and the prevalence of pelvic floor disorder. The chi-squared test was conducted in a bivariate analysis to determine the association between socio-demographic and obstetric characteristics and the prevalence of pelvic floor disorder. Moreover, the multivariate logistic regression test was then conducted in a multivariate analysis. The variables that had correlations with the total pelvic floor distress at *P* < 0.2 were identified as independent variables, whereas the total pelvic floor distress was used as the dependent variable.

## Results

The mean (± SD) of age and the BMI of participants were 34.44 (7.23) and 26.9 (4.15) kg/m2, respectively. Nearly two-fifths of women (42.0%, 168) had high school and diploma education level, whereas one-third of women (28.5%, 114) had academic education. More than four-fifths of women (82.0%, 329) were housekeeper. Moreover, 19 women (4.8%) were nulliparous, and nearly half of women (48.8%, 195) had experienced two deliveries. More than half of women (52.8%, 211) had experienced C-sections. Three women (0.8%) smoked cigarettes. Most of them (95.8%, 383) had no familial history of pelvic floor disorder. Regarding the household income, more than half of participants (57.0%, 228) stated that they had sufficient incomes for livelihood. Nearly two-fifths of women (37.8%,151) did sports exercises, out of whom 88 participants (22.0%) did exercises three times a week. Most of them declared no history of diabetes (99.3%, 397) or chronic respiratory diseases (96.8%, 387). Nearly one-sixth of women (17.0%, 68) had constipation and hemorrhoid. Regarding a history of surgery, only four participants (1.0%) said that they had experienced hysterectomy. None of the women mentioned anything about a history of pelvic prolapse surgery (Table 1).

**Table 1:** Socio-demographic and obstetric characteristic of the participants and their relationships with total pelvic floor disorder (*n* = 400)

Variable	Mean (SD)^a^	Frequency of pelvic floor disorder		Variable	Mean (SD)^a^	Frequency of pelvic floor disorder	
		N (%)^b^	*P*-value^c^			N (%)^b^	*P*-value^c^
Age (Years)	34.44 (7.23)			Body Mass Index (kg/m^2^)	26.9 (4.15)		
	N (%)^b^		0.421		N (%)^b^		0.389
≤ 30	115 (28.7)	83 (27.0)		< 18.5	10 (2.5)	9 (3.0)	
31–40	195 (48.8)	149 (49.0)		18.5–24.9	123 (30.8)	88 (29.0)	
> 40	90 (22.5)	72 (24.0)		25.29.9	184 (46.0)	141 (46.0)	
Education			0.695	≥ 30	83 (20.8)	66 (22.0)	
Elementary	48 (12.0)	34 (11.2)		Income			0.869
Middle school	70 (17.5)	52 (17.1)		Completely Sufficient	74 (18.5)	58 (19.1)	
High school/Diploma	168 (42.0)	132 (43.4)		Relatively sufficient	228 (57.0)	172 (56.6)	
University	114 (28.5)	86 (28.3)		Insufficient	98 (24.5)	74 (24.3)	
Job			0.123	Exercise			0.250
Housekeeper	329 (82.0)	245 (80.6)		Yes	151 (37.8)	110 (36.2)	
Employed outdoors	71 (18.0)	59 (19.4)		No	249 (62.3)	194 (63.8)	
Gravid			0.866	Amount of exercise			
0	19 (4.8)	13 (4.3)		Once a week	36 (9.0)		
1	115 (28.7)	88 (29.0)		Twice a week	27 (6.8)		
2	165 (41.3)	127 (41.8)		Three times a week	88 (22.0)		
≥ 3	101 (25.3)	76 (25.0)		Diabetes Mellitus			0.329
Parity			0.487	Yes	3 (0.8)	3 (1.0)	
0	19 (4.8)	13 (4.3)		No	397 (99.3)	301 (99.0)	
1	138 (34.5)	106 (35.0)		Constipation			< 0.001
2	195 (48.8)	152 (50.0)		Yes	68 (17.0)	64 (21.0)	
≥ 3	48 (12.0)	33 (11.0)		No	332 (83.0)	240 (79.0)	
Type of delivery			0.507	Hemorrhoids			0.097
Vaginal Delivery	138 (34.5)	110 (38.0)		Yes	68 (17.0)	57 (19.0)	
Cesarean Section	211 (52.8)	158 (54.3)		No	332 (83.0)	247 (81.0)	
Both	32 (8.0)	23 (8.0)		History of surgery			
Number of vaginal delivery		0.360	Hysterectomy				0.962
1	69 (17.3)	56 (18.4)		Yes	4 (1.0)	3 (1.0)	
2	77 (19.3)	61 (20.0)		No	396 (99.0)	301 (99.0)	
≥ 3	23 (5.8)	15 (4.9)		Prolapse surgery			-
Smoking			0.329	Yes	-	-	
Yes	3 (0.8)	3 (1.0)		No	400 (100.0)	304 (100.0)	
No	397 (99.3)	301 (99.0)		Chronic lung disease			0.262
Family history of pelvic floor disorders	0.227	Yes	13 (3.3)	12 (4.0)			
Yes	17 (4.3)	15 (5.0)		No	387 (96.8)	292 (96.0)	
No	383 (95.8)	289 (95.0)					

^a^Standard Deviation, ^b^Number (Percent), ^c^Chi-square test

There were 304 (76.0%) participants with pelvic floor disorder. Regarding the subcategories of the pelvic floor distress index, there were 217 (54.3%) participants with pelvic organ prolapse distress 6 (POPDI-6), whereas there were 247 (61.8%) participants with colorectal-anal distress 8 (CRAD-8). Moreover, there were 197 (49.3%) participants with unready distress 6 (UDI-6) (Table 2).

**Table 2:** Prevalence of total pelvic floor disorder and disorder in subcategories (PFDI-20) (*n* = 400)

Variable	Prevalence of disorder number (Percent)
Pelvic Floor Disability Index (PFDI-20)	304 (76.0)
Pelvic Organ prolapse Distress Inventory 6 (POPDI-6)	217 (54.3)
Colorectal-Anal distress Inventory 8 (CRAD-8)	247 (61.8)
Urinary distress Inventory 6 (UDI-6)	197 (49.3)

The chi-squared test indicated that job (*P* = 0.123), constipation (*P* < 0.001), and hemorrhoid (*P* = 0.097) were identified as independent variables correlating with the pelvic floor disorder index in the bivariate analysis with *P* < 0.2. According to the results of multivariate logistic regression, constipation increased the pelvic floor disorder risk by nearly six times (Odds Ratio = 5.62; 95% CI 1.97 to 16.03; *P* = 0.001) (Table 3).

**Table 3:** Related factors with pelvic floor disorder based on the multivariate logistic regression model (*n* = 400)

Variable^b^	Odds ratio (95% CI ^a^)	*P*
Job		
Housekeeper	0.63 (0.32 to 1.26)	0.193
Employed (Reference)	1	
Constipation		
Yes	5.62 (1.97 to 16.03)	0.001
No (Reference)	1	
Hemorrhoids		
Yes	1.37 (0.67 to 2.80)	0.391
No (Reference)	1	

^a^%95 Confidence Interval, ^b^The variables of job, constipation, and hemorrhoid were identified as independent variables associating with the total pelvic floor disorder in the bivariate analysis with *P* < 0.2

## Discussion

According to the results, three-fourths of participants had pelvic floor disorder, and the most prevalent disorder was the colorectal-anal distress. The results also indicated that constipation increased the risk of pelvic floor disorder.

The research findings showed that 76% of women in this study had pelvic floor disorder. The prevalence of pelvic organ prolapse distress, colorectal-anal distress, and urinary distress as subdomains of pelvic floor disorder were 54.3%, 61.8%, and 49.3%, respectively. These prevalence rates are consistent with the findings of a retrospective cohort study conducted by Suemitsu et al*.* (2022) analyzing postnatal women (6 to 15 months after childbirth). In line with the results of the present study, they estimated the prevalence of pelvic floor disorder at 74% through the Japanese version of PDFI-20 [22]. The results were also relatively consistent with the findings reported by Teymouri et al. (2006) who analyzed Iranian women of reproductive age and menopausal age and determined the prevalence of pelvic floor disorder at 69% [23]. This prevalence rate was reported 41% in an Islamic country using the Arabic version of the PDFI-20 through a cross-sectional study on women referring to primary health centers [24]. However, the prevalence reported in the present study is higher than the estimates ranging nearly between 20 and 41% reported in African and American countries. According to those reports, pelvic floor disorder is among the prevalent problems of women all over the world [10, 25, 26]. In a cross-sectional study, MacLennan et al*.* reported that 46% of women had pelvic floor disorder [27]. Another study reported the prevalence of this dysfunction at 37% [28]. Contradictory findings are probably due to differences in demographic information such as age groups, various methodologies, different evaluation tools of pelvic floor disorder, and the level of health care. A middle level of health care of women in Iran [29] and also the high prevalence of inactivity among Iranian women [30] can be mentioned as the possible reasons for the high prevalence of pelvic floor disorders in our study. Given the high prevalence of pelvic floor distress in the present study, it is recommended to conduct screening tests on women in terms of risk factors and use of preventive methods by eliminating risk factors in addition to using pelvic floor retraining programs such as pelvic floor exercises and physical therapy of pelvic floor to improve pelvic floor function [31].

According to the findings of this study, constipation can significantly increase the risk of pelvic floor disorder. In line with this finding, Amselem et al. (2010) introduced constipation as an important risk factor in the emergence of pelvic floor disorder in 31% of cases [32]. Many other studies also introduced constipation as a serious risk factor in the occurrence of pelvic floor disorder [33, 34]. Constipation is the excretion of stiff feces that can harm pelvic floor through pressure exerted on the interior muscles and organs of pelvis while defecation [32, 33]. The total prevalence of constipation was reported 14% in a systematic review. This figure ranges between 5% and 9.9% in Iran. It is more prevalent in women, the elderly, and colon distress cases [35]. According to the results of this systematic review, the high prevalence of colorectal-anal distress can be correlated with constipation, which confirms our finding that the prevalence of colorectal disorders was high among the components of pelvic floor disorders. Hence, patients are first advised to consume fibrous foods (e.g*.*, fruits and vegetables), drink sufficient liquids, and do sports exercises to prevent and cure minor constipation. In serious cases of constipation, patients are advised to take laxatives (e.g*.*, Bisacodyl suppository and magnesium hydroxide syrup), herbal medicine (e.g*.*, Senna leaves), and stool softeners (e.g., Docusate sodium) [36, 37].

According to the evidence from a systematic review and a meta-analysis (2021), vaginal delivery can increase the risk of pelvic floor disorder [17]. This study introduced the increasing number of parities and the BMI as the risk factors in pelvic floor disorder [38]; however, another study reported that pelvic floor disorder had no significant correlations with the number of pregnancies and delivery [39]. Reporting similar findings to those of the present study, an overview indicated that pelvic floor dysfunction had no correlations with social-obstetric variables such as education, occupation, chronic conditions, cigarette smoking, and history of hysterectomy [38]. However, another study introduced aging, number of deliveries, type of delivery, and constipation as the risk factors in the emergence of pelvic floor disorder [40]. According to the evidence, there are contradictory research findings regarding the risk factors of pelvic floor disorder. Due to the effects of this dysfunction on women’s mental health and quality of life [7, 8], it is essential to employ valid tools to measure the prevalence of pelvic floor disorder and its relevant risk factors in an effort to reduce the prevalence of this disorder by eliminating its risk factors.

The strengths of this study include the use of a valid tool to evaluate pelvic floor disorder [41] and random selection of participants at reproductive age. However, since the statistical population included women of reproductive age with Azeri ethnicity, there might be certain limitations on generalization of results to other Iranian ethnicities and menopausal women. Another limitation is due to the nature of cross-sectional studies; the relationships found between pelvic floor disorder and constipation does not accurately indicate a causal relationship. Therefore, it is suggested to conduct similar studies on other Iranian ethnicities and other age groups including menopausal women and also to conduct more studies with stronger design such as cohort studies to determine the risk factors related to pelvic floor disorder.

This study reported the high prevalence of pelvic floor disorder, which is an important health issue affecting women’s quality of life. The study also introduced constipation as a risk factor. Therefore, it is possible to prevent this condition and decelerate its progress by adopting nutritional solutions, moderating lifestyles, and conducting timely identification and screening tests through valid questionnaires.

## Conclusion

According to the study findings, pelvic floor disorder is highly prevalent among Iranian women of reproductive age. Moreover, constipation was introduced as a risk factor. Thus, it is recommended to perform screening tests through valid tools and offer preventive measures for the prevention and cure of constipation to reduce the prevalence of pelvic floor disorder.

## Data Availability

The datasets generated and/or analyzed during the current study are not publicly available due to the limitations of ethical approval involving the patient data and anonymity, but are available from the corresponding author upon reasonable requests.

## Abbreviations

PDFI-20: Pelvic floor distress inventory-20
U/A: Urine analysis
POPDI-6: Pelvic organ prolapse distress 6
CRAD-8: Colorectal-anal distress 8
UDI-6: Unready distress 6
ICC: Intraclass correlation coefficient
BMI: Body mass index

## References

[1] Memon HU, Handa VL (2013). Vaginal childbirth and pelvic floor disorders. Womens health.

[2] Boyle R, Hay-Smith EJC, Cody JD, Mørkved S (2014). Pelvic floor muscle training for prevention and treatment of urinary and fecal incontinence in antenatal and postnatal women: a short version Cochrane review. Neurourol Urodyn.

[3] Turner CE, Young JM, Solomon MJ, Ludlow J, Benness C (2009). Incidence and etiology of pelvic floor dysfunction and mode of delivery: an overview. Dis Colon Rectum.

[4] Haylen BT, De Ridder D, Freeman RM, Swift SE, Berghmans B, Lee J (2010). An international urogynecological association (IUGA)/international continence society (ICS) joint report on the terminology for female pelvic floor dysfunction. Neurourol Urodyn: Off J Int Continence Soc.

[5] Norton C, Whitehead W, Bliss D, Harari D, Lang J (2010). Management of fecal incontinence in adults. Neurourol Urodyn: Off J Int Continence Soc.

[6] Wein AJ (2015). Re: lifetime risk of stress urinary incontinence or pelvic organ prolapse surgery. J Urol.

[7] Good MM, Solomon ER (2019). Pelvic floor disorders. Obstet Gynecol Clin.

[8] Meekins AR, Siddiqui NY (2020). Diagnosis and management of postpartum pelvic floor disorders. Obstet Gynecol Clin.

[9] Sánchez-Sánchez B, Torres-Lacomba M, Yuste-Sánchez MJ, Navarro-Brazález B, Pacheco-da-Costa S, Gutiérrez-Ortega C (2013). Cultural adaptation and validation of the pelvic floor distress inventory short form (PFDI-20) and pelvic floor impact questionnaire short form (PFIQ-7) Spanish versions. Eur J Obstet Gynecol Reprod Biol.

[10] Dheresa M, Worku A, Oljira L, Mengiste B, Assefa N, Berhane Y (2018). One in five women suffer from pelvic floor disorders in Kersa district Eastern Ethiopia: a community-based study. BMC womens health.

[11] Megabiaw B, Adefris M, Rortveit G, Degu G, Muleta M, Blystad A (2013). Pelvic floor disorders among women in Dabat district, northwest Ethiopia: a pilot study. Int Urogynecol J.

[12] Eftekhar T, Ghanbari Z, Kalantari F, Shariat M, Haghollahi F (2012). The frequency of pelvic floor dysfunctions and their risk factors in women aged 40–55. J Family Reprod Health..

[13] Yang X, Zhu L, Li W, Sun X, Huang Q, Tong B (2019). Comparisons of electromyography and digital palpation measurement of pelvic floor muscle strength in postpartum women with stress urinary incontinence and asymptomatic parturients: a cross-sectional study. Gynecol Obstet Invest.

[14] Hallock JL, Handa VL (2016). The epidemiology of pelvic floor disorders and childbirth: an update. Obstet Gynecol Clin.

[15] Hage‐Fransen MA, Wiezer M, Otto A, Wieffer‐Platvoet MS, Slotman MH, Nijhuis‐van der Sanden MW, et al. Pregnancy‐and obstetric‐related risk factors for urinary incontinence, fecal incontinence, or pelvic organ prolapse later in life: a systematic review and meta‐analysis. Acta Obstet Gynecol Scand. 2021;100(3): 373–82. 10.1111/aogs.14027 33064839

[16] Weintraub AY, Glinter H, Marcus-Braun N (2019). Narrative review of the epidemiology, diagnosis and pathophysiology of pelvic organ prolapse. Int Braz J Urol.

[17] Barca JA, Bravo C, Pintado-Recarte MP, Asúnsolo Á, Cueto-Hernández I, Ruiz-Labarta J (2021). Pelvic floor morbidity following vaginal delivery versus cesarean delivery: systematic review and meta-analysis. J Clin Med.

[18] López-López AI, Sanz-Valero J, Gómez-Pérez L, Pastor-Valero M (2021). Pelvic floor: vaginal or caesarean delivery? A review of systematic reviews. Int Urogynecol J.

[19] Nygaard IE, Wolpern A, Bardsley T, Egger MJ, Shaw JM (2021). Early postpartum physical activity and pelvic floor support and symptoms 1 year postpartum. Am J Obstet Gynecol.

[20] Dieter AA, Wilkins MF, Wu JM (2015). Epidemiological trends and future care needs for pelvic floor disorders. Curr Opin Obstet Gynecol.

[21] Barber M, Walters M, Bump R (2005). Short forms of two condition-specific quality-of-life questionnaires for women with pelvic floor disorders (PFDI-20 and PFIQ-7). Am J Obstet Gynecol.

[22] Suemitsu T, Mikuni K, Matsui H, Suzuki M, Takahashi T. Prevalence and risk factors of pelvic floor disorders after delivery in Japanese women using the PFDI-20: a single-center retrospective cohort survey. Preprint at 10.21203/rs.3.rs-1678303/v1(2022). 10.7759/cureus.40152 PMC10250108 37304387

[23] Taimoori B, Roudbari M (2006). The prevalence of symptoms of pelvic floor disorders in women that referred to the clinic of gynecology in Ali-ebn-Abitaleb hospital, Zahedan. Iran. Zahedan J Res Med Sci.

[24] Saudi RA, Tosson EE (2022). Prevalence and the degree of distress of pelvic floor disorders symptoms in women seeking primary health care at Ismailia governorate. Egypt Urogynaecologia.

[25] Minassian VA, Drutz HP, Al-Badr A (2003). Urinary incontinence as a worldwide problem. Int J Gynaecol Obstet.

[26] Beketie ED, Tafese WT, Assefa ZM, Berriea FW, Tilahun GA, Shiferaw BZ (2021). Symptomatic pelvic floor disorders and its associated factors in South-Central Ethiopia. PLoS ONE.

[27] MacLennan AH, Taylor AW, Wilson DH, Wilson D (2000). The prevalence of pelvic floor disorders and their relationship to gender, age, parity and mode of delivery. BJOG.

[28] Lukacz ES, Lawrence JM, Contreras R, Nager CW, Luber KM (2006). Parity, mode of delivery, and pelvic floor disorders. Obstet Gynecol.

[29] Sheikhbardsiri H, Abdar ZE, Sheikhasadi H, Mahani SA, Sarani A (2020). Observance of patients’ rights in emergency department of educational hospitals in south-east Iran. Int J Hum Rights Healthc.

[30] Fakhrzadeh H, Djalalinia S, Mirarefin M, Arefirad T, Asayesh H, Safiri S (2016). Prevalence of physical inactivity in Iran: a systematic review. J Cardiovasc Thorac Res.

[31] Badakhsh M, Khorasani B, Arab AM, Forootan M (2014). The role of pelvic floor muscle dysfunction in subjects with fecal incontinence and efficacy of pelvic floor muscle retraining in treatment: a literature review. Govaresh.

[32] Amselem C, Puigdollers A, Azpiroz F, Sala C, Videla S, Fernandez-Fraga X (2010). Constipation: a potential cause of pelvic floor damage?. Neurogastroenterol Motil.

[33] Sangsawang B (2014). Risk factors for the development of stress urinary incontinence during pregnancy in primigravidae: a review of the literature. Eur J Obstet Gynecol Reprod Biol.

[34] Bradley CS, Kennedy CM, Turcea AM, Rao SS, Nygaard IE (2007). Constipation in pregnancy: prevalence, symptoms, and risk factors. Obstet Gynecol.

[35] Suares NC, Ford AC (2011). Prevalence of, and risk factors for, chronic idiopathic constipation in the community: systematic review and meta-analysis. ACG.

[36] Rao SS, Patcharatrakul T (2016). Diagnosis and treatment of dyssynergic defecation. J Neurogastroenterol Motil.

[37] Chatoor D, Emmnauel A (2009). Constipation and evacuation disorders. Best Pract Res Clin Gastroenterol.

[38] Walters MD. Pelvic floor disorders in women: an overview. Rev Med Univ Navarra. 2004;48(4):9–12, 15–7. 15810715

[39] Sleep J, Grant A, Garcia J, Elbourne D, Spencer J, Chalmers I (1984). West Berkshire perineal management trial. Br Med J (Clin Res Ed).

[40] Kepenekci I, Keskinkilic B, Akinsu F, Cakir P, Elhan AH, Erkek AB (2011). Prevalence of pelvic floor disorders in the female population and the impact of age, mode of delivery, and parity. Dis Colon Rectum.

[41] Barber MD, Kuchibhatla MN, Pieper CF, Bump RC (2001). Psychometric evaluation of 2 comprehensive condition-specific quality of life instruments for women with pelvic floor disorders. Am J Obstet Gynecol.

